# Extracellular vesicular Wnt7b mediates HPV E6-induced cervical cancer angiogenesis by activating the β-catenin signaling pathway

**DOI:** 10.1186/s13046-020-01745-1

**Published:** 2020-11-25

**Authors:** Jun-Jun Qiu, Shu-Gen Sun, Xiao-Yan Tang, Ying-Ying Lin, Ke-Qin Hua

**Affiliations:** 1 Department of Gynaecology, Obstetrics and Gynaecology Hospital, Fudan University, 419 Fangxie Road, Shanghai, 200011 China; 2 Shanghai Key Laboratory of Female Reproductive Endocrine-Related Diseases, 413 Zhaozhou Road, Shanghai, 200011 China; 3 Department of Neurosurgery, Ren Ji Hospital, School of Medicine, Shanghai Jiao Tong University, 160 Pujian Road, Shanghai, 200127 China

**Keywords:** Cervical cancer, Human papillomavirus, E6, Extracellular vesicles, Wnt7b

## Abstract

**Background:**

The E6 oncoproteins of human papillomavirus (HPV) 16/18 are the critical drivers of cervical cancer (CC) progression. Extracellular vesicles (EVs) are emerging as critical mediators of cancer-tumor microenvironment (TME) communication. However, whether EVs contribute to HPV 16/18 E6-mediated impacts on CC progression remains unclear.

**Methods:**

A series of in vitro and in vivo assays were performed to elucidate the roles and mechanism of EV-Wnt7b in HPV E6-induced CC angiogenesis. The prognostic value of serum EV-Wnt7b was determined and a predictive nomogram model was established.

**Results:**

HPV 16/18 E6 upregulated Wnt7b mRNA expression in four HPV 16/18-positive CC cell lines and their EVs. In vitro and in vivo experiments demonstrated that EV-Wnt7b mRNA was transferred to and modulated human umbilical vein endothelial cells (HUVECs) toward more proliferative and proangiogenic behaviors by impacting β-catenin signaling. Clinically, serum EV-Wnt7b levels were elevated in CC patients and significantly correlated with an aggressive phenotype. Serum EV-Wnt7b was determined to be an independent prognostic factor for CC overall survival (OS) and recurrence-free survival (RFS). Notably, we successfully established a novel predictive nomogram model using serum EV-Wnt7b, which showed good prediction of 1- and 3-year OS and RFS.

**Conclusions:**

Our results illustrate a potential crosstalk between HPV 16/18-positive CC cells and HUVECs via EVs in the TME and highlight the potential of circulating EV-Wnt7b as a novel predictive biomarker for CC prognosis.

**Supplementary Information:**

The online version contains supplementary material available at 10.1186/s13046-020-01745-1.

## Introduction

Despite the development of screening tests and therapies, cervical cancer (CC) remains one of the most lethal gynecological malignances worldwide, especially in developing countries [1]. Persistent infection with high-risk human papillomaviruses (HPVs), including HPV 16 and 18, is the most crucial cause of CC development [2, 3]. Additionally, the carcinogenic potential of HPV 16/18 is mainly due to the expression of the E6 and E7 proteins, which dysregulate p53 and pRb, respectively, and are critical for the transformation and maintenance of the malignant phenotype [4, 5]. Despite these findings, however, HPV infection alone is inadequate for CC carcinogenesis and progression because not all patients infected with HPV eventually develop CC. Thus, additional cofactors, such as the tumor microenvironment (TME), must be implicated in HPV E6/E7-induced CC progression.

Recently, increasing evidence has demonstrated that the TME is not only a consequence of but also a contributor to cancer progression. In the TME, various components, including immune cells, fibroblasts, the extracellular matrix, and endothelial cells, play important roles in regulating immune escape, tumor proliferation, metastasis and angiogenesis [6–10]. Of note, angiogenesis has been widely accepted as a key event in tumor development because cancer cells rely on neovascularization for nutrition and oxygen supply to support continuous and sustained growth and metastasis [11]. In CC, angiogenesis represents a critical contributor in controlling the shift from dysplasia to aggressive CC [12, 13]. Although previous studies have implicated HPV in angiogenesis [14, 15], the detailed cellular and molecular events involved, especially how HPV mediates cancer-TME communication to influence angiogenesis, remain unclear.

Currently, extracellular vesicles (EVs), small bilayer membrane-bound nanoparticles that are 30–1000 nm in diameter, are established as critical mediators of cell-cell communication within the TME [16–18]. EVs can modulate recipient cells to remodel the TME through the transfer of their cargos, including DNAs, RNAs, proteins and signaling molecules [19–23]. Among the cargos, Wnt signaling molecules attract particular attention because Wnt proteins are powerful angiogenic factors [24], and the activity of related signaling pathways is regarded as a critical requirement to initiate HPV E6-induced CC malignant transformation [25, 26]. Previously, EV-mediated transfer of Wnt 11, Wnt3a, Wnt5b, and Wnt4 has been described in the progression of various cancers [27–30]. However, in HPV-associated CC, whether cancer-derived EVs can secrete and transport Wnt and how these EVs communicate with the TME to influence angiogenesis remain largely unknown.

In the present study, we explored the unique crosstalk between HPV 16/18-positive CC cells and human umbilical vein endothelial cells (HUVECs) via EVs in the TME. Notably, we proposed a novel mechanism for HPV 16/18 E6-induced CC progression from the perspective of “EV-shuttled Wnt7b activating β-catenin signaling” and highlighted the potential of circulating EV-Wnt7b as a predictive biomarker for CC prognosis.

## Materials and methods

### Patients and samples

We collected serum samples from 101 squamous cervical cancer (SCC) patients who underwent surgery at the Obstetrics and Gynecology Hospital of Fudan University between December 2013 and October 2014. All SCC specimens were confirmed by pathology. Patients who received radiotherapy/chemotherapy before surgery or had any other diseases were excluded. Thirty control serum samples were collected from healthy participants who underwent routine physical examination. All specimens were immediately stored at − 80 °C in our tissue bank until use. The clinicopathological characteristics of the patients, including age, International Federation of Gynecology and Obstetrics (FIGO) stage, tumor size, lymphovascular invasion, stromal invasion depth, lymph node metastasis, parametrial invasion, margin, are detailed in Supplementary Table 1. Overall survival (OS) was calculated from the date of surgery to the date of death or the end of follow-up. Recurrence-free survival (RFS) was defined as the time interval between the date of surgery to the date of recurrence or the end of follow-up.

### Cells and cultures

Five human CC cell lines (HPV 16-positive SiHa and CaSki cells, HPV 18-positive HeLa and SW756 cells, and HPV-negative C33A cells) were obtained from the American Type Culture Collection (Manassas, VA, USA). All cell lines were authenticated using short tandem repeat (STR) profiling. All mycoplasma-free cells were cultured at 37 °C in a humidified incubator with 5% CO_2_ and grown in Eagle’s minimum essential medium (EMEM; Siha, HeLa and C33A cells), RPMI-1640 medium (Caski cells), or Leibovitz’s L-15 medium (SW756 cells) (Invitrogen; Thermo Fisher Scientific, Inc.) supplemented with 10% fetal bovine serum (Gibco; Thermo Fisher Scientific, Inc.), 2 mM L-glutamine, penicillin (100 units/ml), and streptomycin (100 μg/ml).

### Establishment of stable knockdown (KD) or overexpression (OE) cell lines

For stable KD of the E6 oncogene, E6-shRNAs were constructed by targeting the sequences of HPV 16 with KD-1: 5′-GGTCGATGTATGTCTTGTTGC-3′; KD-2: 5′-GGGAATCCATATGCTGTATGT-3′; and KD-3: 5′-GCTGCAAACAACTATACATGA-3′; and those of HPV 18 with KD-1: 5′-GGTGCCAGAAACCGTTGAATC-3′; KD-2: 5′-ACCCTACAAGCTACCTGATCT-3′; and KD-3: 5′-GACTCCAACGACGCAGAGAAA-3′. Lentiviruses encoding E6-KD or a negative control (NC) were generated by Hanyin Co. (Shanghai, China). To stably overexpress Wnt7b, the full-length Wnt7b gene was synthesized and cloned into a lentiviral vector (named Wnt7b-OE); the control viruses were named Wnt7b-NC. After infection with the lentiviruses and selection for 2 weeks using puromycin, stable cell lines were established. The efficiency of KD or OE was confirmed by qRT-PCR.

### Quantitative real-time polymerase chain reaction (qRT-PCR)

RNA extraction and qRT-PCR experiments were performed as previously described [31]. The primer sequences of Wnt1, Wnt2, Wnt3a, Wnt4, Wnt5a, Wnt6, Wnt7b, Wnt10b, Wnt11, HPV16-E6, HPV18-E6 and GAPDH are listed in Supplementary Table 2. The relative expression of each gene was calculated based on the corresponding Ct values, normalized to Glyceraldehyde-3-phosphate dehydrogenase (GAPDH) expression. Fold changes in the expression of each gene were calculated by a comparative Ct method using the 2^-ΔΔCt^ relative quantification method.

### Western blotting

We performed Western blotting as previously described [32]. Primary antibodies against Wnt7b, β-catenin, CD9, CD63 and TSG101 were purchased from Abcam (Cambridge, UK). Antibodies against Flag and Hsp70 were purchased from Cell Signaling Technology (Massachusetts, US).

### Isolation and verification of EVs

MISEV2018 recommendations were followed for EV isolation. After centrifuging serum free cell culture supernatants or serum samples, we used the total EV isolation kit (catalogue no. EXOTC50A-1; System Biosciences, Palo Alto, California) to isolate EVs from the cell supernatants (30 ml/48 h) and used the ExoQuick EV Precipitation Solution Kit (catalog no. EXOQ5A-1; System Biosciences, Palo Alto, CA, USA) to isolate EVs from the serum samples (5 ml) according to the protocol. The concentration of EVs was tested by a BCA assay. The EVs were stored at − 80 °C until use.

Three methods including transmission electron microscopy (TEM), nanoparticle tracking analysis (NTA) and Western blotting were used to verify the isolated EVs as previously described [33]. Briefly, EV suspension samples were observed under a Hitachi H-7650 transmission electron microscope (Hitachi, Ltd., Tokyo, Japan) for the TEM analysis. Additionally, resuspended EVs were detected using a NanoSight NS300 instrument (Malvern Instruments Ltd., Worcestershire, UK) to test the size distribution of the EVs. Moreover, known EV biomarkers were tested by Western blotting for CD9, TSG101, Hsp70 and CD63.

### The internalization of labelled EVs by the HUVECs

To track the internalization of EVs, EVs were labelled with PKH67 (Sigma-Aldrich; Merck KGaA, Darmstadt, Germany) as previously described [33, 34]: EVs resuspended in a buffer provided in the kits were mixed with the PKH67 dyes and were incubated for 5 min at room temperature. Next, the samples were added to PBS supplemented with 5% bovine serum albumin and were ultracentrifuged at 100,000 g for 1 h to remove free dyes. The labelled EVs were co-cultured with HUVECs for 6 h, which were then fixed with 4% paraformaldehyde at 25 °C for 20 min. The internalization of labelled EVs by the HUVECs was analysed using a fluorescence confocal microscope. All the fluorescence images were captured.

### Cell counting Kit-8 (CCK8) proliferation assay

We conducted cell proliferation assays using CCK8 (Dojindo, Japan). The cell numbers per well were then determined by testing the absorbance (450 nm) using a 96-well plate reader at the indicated time points.

### Tube formation assay

A tube formation assay was conducted as previously described [33]. Briefly, EV-pretreated cells were incubated with serum-free medium for 12 h and then transferred into 48-well plates precoated with Matrigel. After incubation, tube formation was observed under a microscope. Total tube length was tested by measuring the branches of blood vessels using ImageJ software.

### TOP/FOP luciferase reporter assay

The transcriptional activity of β-catenin was assessed using the TOP/FOP dual-luciferase reporter system (Dual-Glo™ Luciferase Assay System, Promega). The Renilla luciferase plasmid pRLTK (Promega), which controls for transfection efficiency, was cotransfected with β-catenin-responsive firefly luciferase reporter plasmid Top*Flash* (EMD Millipore) or the negative control Fop*Flash* (EMD Millipore) using the lipofectamine 2000 (Thermo Fisher Scientific). Cells were harvested after 24 h in culture and the luciferase activity was determined by the Luciferase Assay System (Promega) using a Microplate Luminometer (Berthold, Bad Wildbad, Germany).

### Nude mouse model

Female BALB/c athymic nude mice, 4–6 weeks old and weighing 20–22 g, were purchased from Slac Laboratory Animal Co., Ltd. (Shanghai, China). The care for animals was in accordance with institution guidelines. SiHa cells (5 × 10^6^) were subcutaneously injected into mice. Twenty-four mice were then injected with EVs or PBS and divided into four groups: (1) PBS group, (2) EV/NC group, (3) EV/E6-KD group, and (4) EV/E6-KD + Wnt7b-OE group. To further investigate the role of Wnt7b/β-catenin signaling, another 18 mice were divided into three groups: (1) EV/E6-KD group, (2) EV/E6-KD + Wnt7b-OE group, and (3) EV/E6-KD + Wnt7b-OE + FH535 group. After four injections with 0.2 mL PBS containing 50 μg EVs, all the mice were euthanized, and the tumors were collected.

### Immunohistochemistry (IHC)

IHC assay was conducted as previously described [33]: Briefly, the density of blood vessels was detected using CD31 staining. Xenograft tumours were fixed, embedded in paraffin and sectioned into 4μm thick slices. After deparaffinization and rehydration, sections were blocked and incubated with a CD31 antibody (Abcam, USA). 

### Statistical analysis

Statistical analysis was performed with SPSS 16.0 (SPSS Inc., Chicago, IL, USA) and GraphPad Prism 6.0 (GraphPad Software, La Jolla, CA, USA). Independent *t*-tests were used to analyze continuous data, and the chi-square test or Fisher’s exact test was used to analyze categorical data. Differences among the groups were analyzed using Student two-tailed *t*-test or one way analysis of variance (ANOVA). OS and RFS analyses were performed using the Kaplan-Meier method, log-rank test, univariate and multivariate Cox regression models. Based on the results of the multivariate Cox analysis, a predictive nomogram for OS and RFS was established using R version 3.5.3 (https://www.r-project.org/) as described in previous studies [33]. *P* < 0.05 was considered statistically significant.

### Ethics committee approval and patient consent

This study was approved by the Research Ethics Committee of the Obstetrics and Gynecology Hospital of Fudan University, China. All samples were obtained with full and informed consent.

## Results

### Wnt7b is highly expressed in HPV 16/18-positive CC cells and downregulated by E6 knockdown

**Fig. 1:**
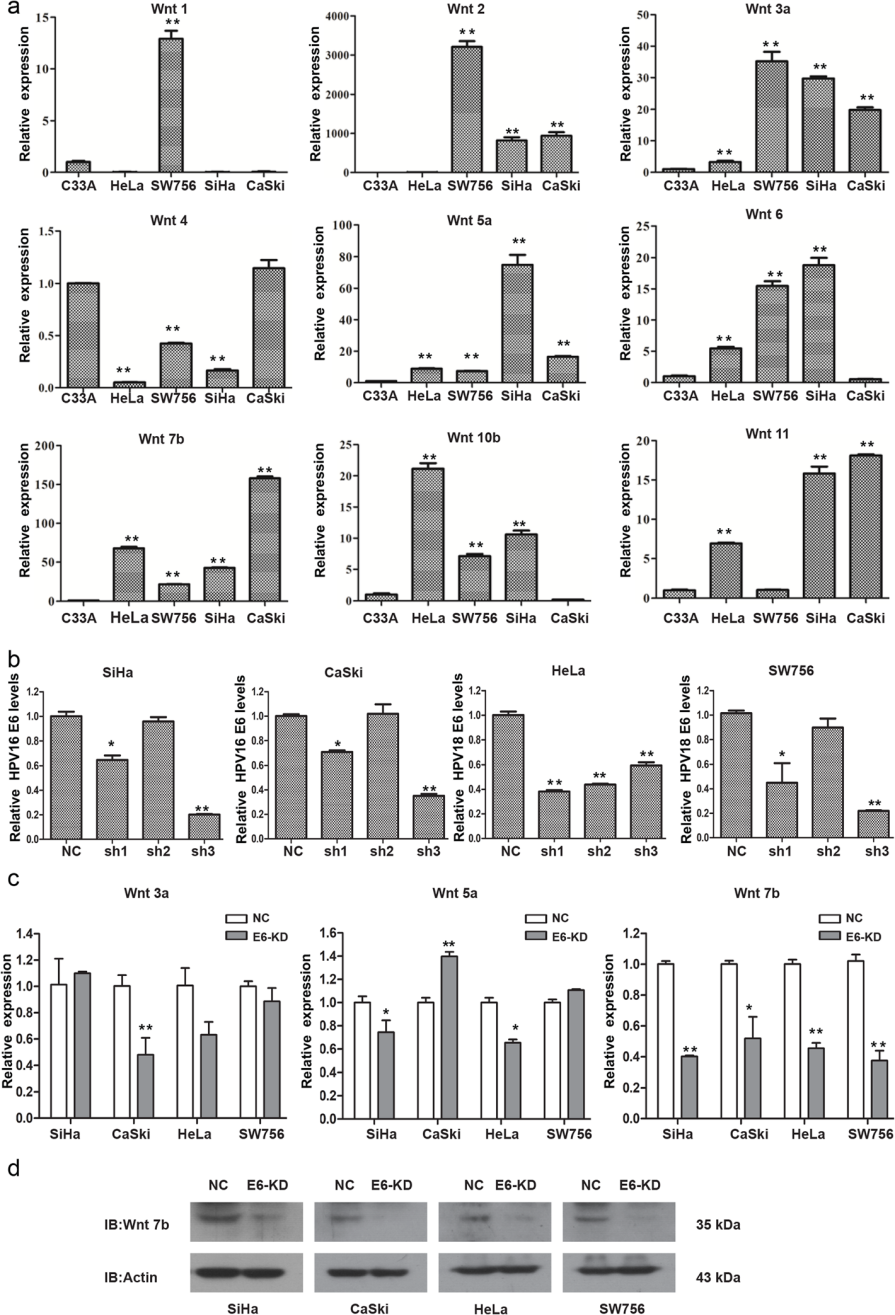
Wnt7b is highly expressed in HPV 16/18-positive CC cells and downregulated by E6 knockdown. **a** The expression of Wnt1, Wnt2, Wnt3a, Wnt4, Wnt5a, Wnt6, Wnt7b, Wnt10b and Wnt11 in CC cells was tested using qRT-PCR. The mRNA levels of Wnt3a, Wnt5a and Wnt7b were significantly increased in all the four HPV-positive cell lines (SiHa and CaSki cells are HPV 16 positive, while HeLa and SW756 cells are HPV 18 positive) compared to HPV-negative C33A cells. **b** The knockdown efficiency of HPV 16/18 E6-shRNAs in the four HPV 16/18-positive CC cell lines was analyzed using qRT-PCR. Based on the different inhibitory effects observed, we constructed stable HPV 18 E6-KD HeLa cells using HPV 18 E6-shRNA1, stable HPV 18 E6-KD SW756 cells using HPV 18 E6-shRNA3, and stable HPV 16 E6-KD SiHa and CaSki cells using HPV 16 E6-shRNA3 in the following study. **c** qRT-PCR analysis showed that the knockdown (KD) of HPV-E6 did not lead to a consistent expression change of Wnt3a and Wnt5a mRNA but lead to a consistently decreased Wnt7b mRNA expression in all the four HPV 16/18 positive cell lines. **d** Western blotting analysis showed the decreased expression of Wnt7b protein by E6-KD in all the four HPV 16/18 positive cell lines. * *P* < 0.05, ** *P* < 0.01

The expression patterns of Wnt1, Wnt2, Wnt3a, Wnt4, Wnt5a, Wnt6, Wnt7b, Wnt10b and Wnt11 in CC cells were tested using qRT-PCR. Notably, we observed consistent higher levels of Wnt3a, Wnt5a and Wnt7b mRNA in all the four HPV 16/18-positive cell lines compared to HPV-negative C33A cells (Fig. 1a), suggesting that Wnt3a, Wnt5a and Wnt7b mRNA may be both HPV 16 and 18-associated.

Since the Wnt pathway can be activated by the HPV E6 oncogene, a prerequisite for CC carcinogenesis, we determined whether Wnt3a, Wnt5a and Wnt7b mRNA can be dysregulated by HPV 16/18 E6. Based on the different inhibitory effects of E6-shRNAs on the four HPV-positive CC cell lines (Fig. 1b), we constructed stable HPV E6-KD HeLa cells using shRNA1 and E6-KD SiHa, CaSki and SW756 cells using shRNA3. We found that the knockdown (KD) of HPV-E6 did not lead to a consistent expression change of Wnt3a and Wnt5a mRNA but lead to a consistently decreased Wnt7b mRNA expression in all the four HPV 16/18 positive cell lines (Fig. 1c). Moreover, the western blotting results further confirmed the decreased expression of Wnt7b protein by E6-KD in all the four HPV 16/18 positive cell lines (Fig. 1d). All these observations suggest that Wnt7b may be specifically upregulated by both HPV 16 and HPV 18 E6 oncogene. Therefore, we focused on Wnt7b for further study.

### EV-Wnt7b mRNA is highly expressed in HPV 16/18-positive CC cells and decreased by E6 knockdown

**Fig. 2:**
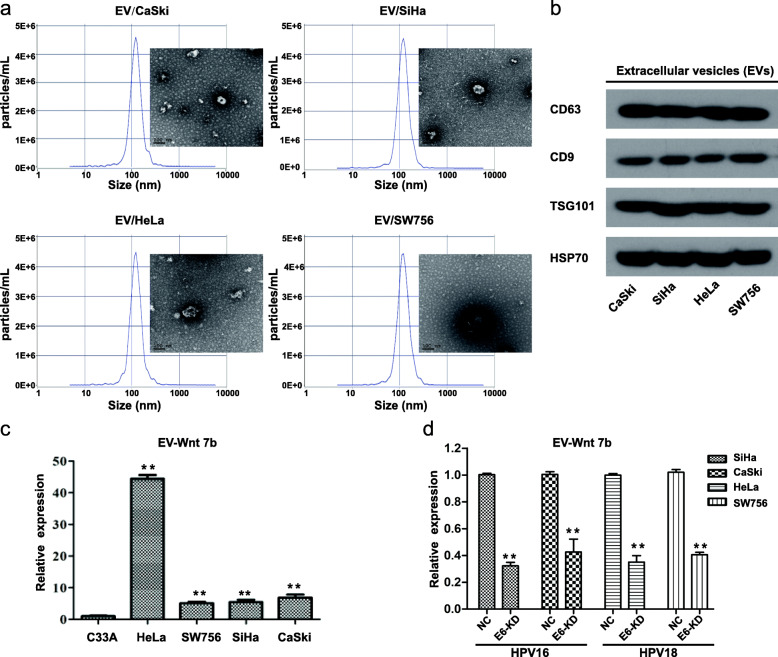
EV-Wnt7b mRNA is highly expressed in HPV 16/18-positive CC cells and decrease by E6 knockdown. **a** Transmission electron microscopy and nanoparticle tracking analysis were used to evaluate CC-derived EVs. The EVs derived from CaSki, SiHa, HeLa and SW756 cells exhibited similar typical round morphologies, with particles ranging from 50 to 150 nm in diameter. **b** Western blot analysis showed positive expression of the known EV markers CD63, CD9, TSG101 and HSP 70 in EVs derived from CaSki, SiHa, HeLa or SW756 cells. **c** qRT-PCR analysis was used to assess Wnt7b in CC-derived EVs. Wnt7b mRNA levels were significantly increased in in EVs derived from 4 HPV 16/18-positive cell lines (the CaSki, SiHa, HeLa and SW756 cell lines) compared to EVs from HPV-negative C33A cells. **d** qRT-PCR analysis showed that E6 knockdown decreases Wnt7b mRNA loading in HPV 16/18 positive cell-derived EVs. * *P* < 0.05, ** *P* < 0.01

To further examine the expression pattern of Wnt7b in CC-derived EVs, we isolated EVs derived from SiHa, CaSki, HeLa and SW756 cells and characterized them using TEM, NTA and Western blotting. The CC-derived EVs appeared to be typical round particles ranging from 50 to 150 nm in diameter. Western blot analysis confirmed the presence of four known EV markers, CD63, CD9, TSG101 and HSP70. These results indicated the effective isolation of EVs (Figs. 2a and b). Further qRT-PCR results showed that Wnt7b mRNA levels were significantly increased in HPV 16/18-positive cell-derived EVs compared to HPV negative cell-derived EVs (Fig. 2c). Moreover, after extracting EVs from HPV 16/18 E6-KD cells and corresponding control cells, we observed that EV-Wnt7b mRNA levels were significantly decreased by E6-KD (Fig. 2d). These results suggest that E6 knockdown decreases Wnt7b mRNA loading in HPV 16/18 positive cell-derived EVs, which is consistent with the cellular results.

Here, we also tested Wnt7b protein in CC cells and their centrifuged EVs-free conditioned medium. We observed that Wnt7b protein was absent or with very low expression in the centrifuged EVs-free conditioned medium, but with significantly high expression in all the four HPV 16/18-positive cell lines compared to HPV-negative C33A cells (Supplementary Fig. 1).

### E6-regulated EV-Wnt7b mRNA can be transferred to HUVECs

**Fig. 3:**
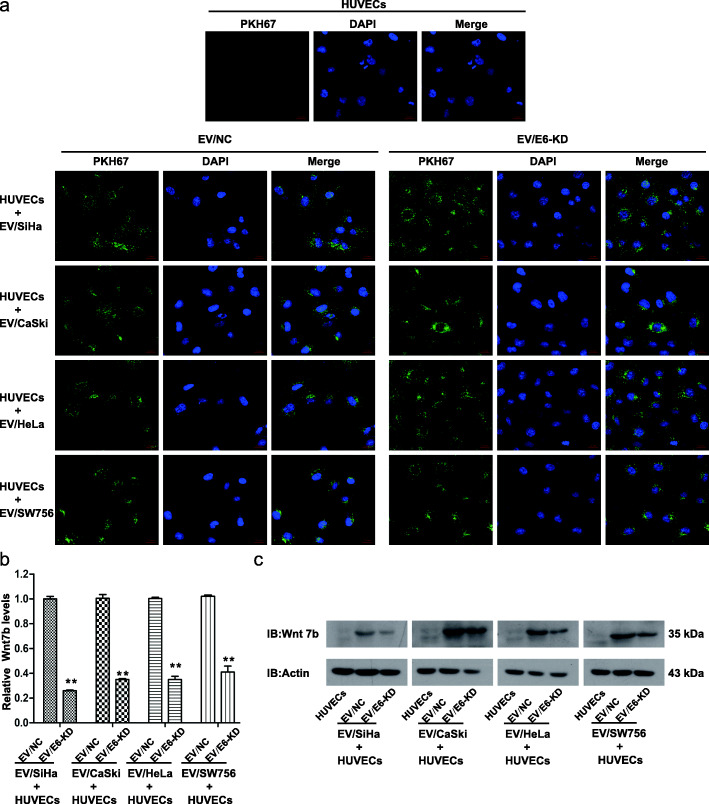
Extracellular vesicular Wnt7b can be transferred to HUVECs. **a** The internalization of CC-derived EVs by HUVECs was evaluated using a fluorescence confocal microscope. PKH67 (green fluorescent dye)-labeled EVs derived from HPV 16 E6-KD cells (SiHa or CaSki cells), HPV 18 E6-KD cells (HeLa or SW756 cells) or their corresponding control cells (EV/NC) were all effectively taken up by HUVECs. Untreated HUVECs were used as the negative control. **b** qRT-PCR analysis showed that Wnt7b mRNA levels were significantly lower in HUVECs treated with EV/E6-KD than in those treated with EV/NC derived from the four HPV 16/18-positive cell lines. **c** Western blot analysis showed very low Wnt7b protein expression in untreated HUVECs. Wnt7b protein levels were increased when treated with EV/NC derived from the four HPV 16/18-positive CC cell lines, while the treatment of EV/E6-KD inhibited such promotion of Wnt7b protein expression. * *P* < 0.05, ** *P* < 0.01

In the TME, cancer-derived EVs can be internalized by recipient cells to achieve their functions. Given that angiogenesis is essential in HPV E6-induced CC progression, we initially sought to investigate whether EVs can mediate HPV E6-induced CC angiogenesis. To this end, we labeled EVs derived from the four HPV 16/18 E6-KD cells (EV/E6-KD) or their corresponding control cells (EV/NC) with PKH67 and observed that all labeled EVs (EV/E6-KD and EV/NC) were effectively internalized by HUVECs using a fluorescence confocal microscope (Fig. 3a). Further qRT-PCR results confirmed that Wnt7b mRNA levels were significantly lower in HUVECs treated with EV/E6-KD than in those treated with EV/NC derived from the four HPV 16/18-positive cell lines (Fig. 3b). These findings suggest that by means of EVs, E6-regulated Wnt7b mRNA can be transported from HPV 16/18-positive CC cells to HUVECs.

Moreover, we also observed very low Wnt7b protein expression in untreated HUVECs. Wnt7b protein levels were increased when treated with EV/NC derived from the four HPV 16/18-positive CC cell lines, while the treatment of EV/E6-KD inhibited such promotion of Wnt7b protein expression (Fig. 3c). Considering these findings, we may infer that Wnt7b mRNA transferred by EVs in HUVEC cells may be followed by Wnt7b protein synthesis in the cells, which may consequently exert effect on HUVECs. Admittedly, we cannot exclude the partial transportation of Wnt7b protein to HUVECs.

### EV-Wnt7b mediates the HPV 16/18 E6-induced proliferation and angiogenesis of HUVECs

**Fig. 4:**
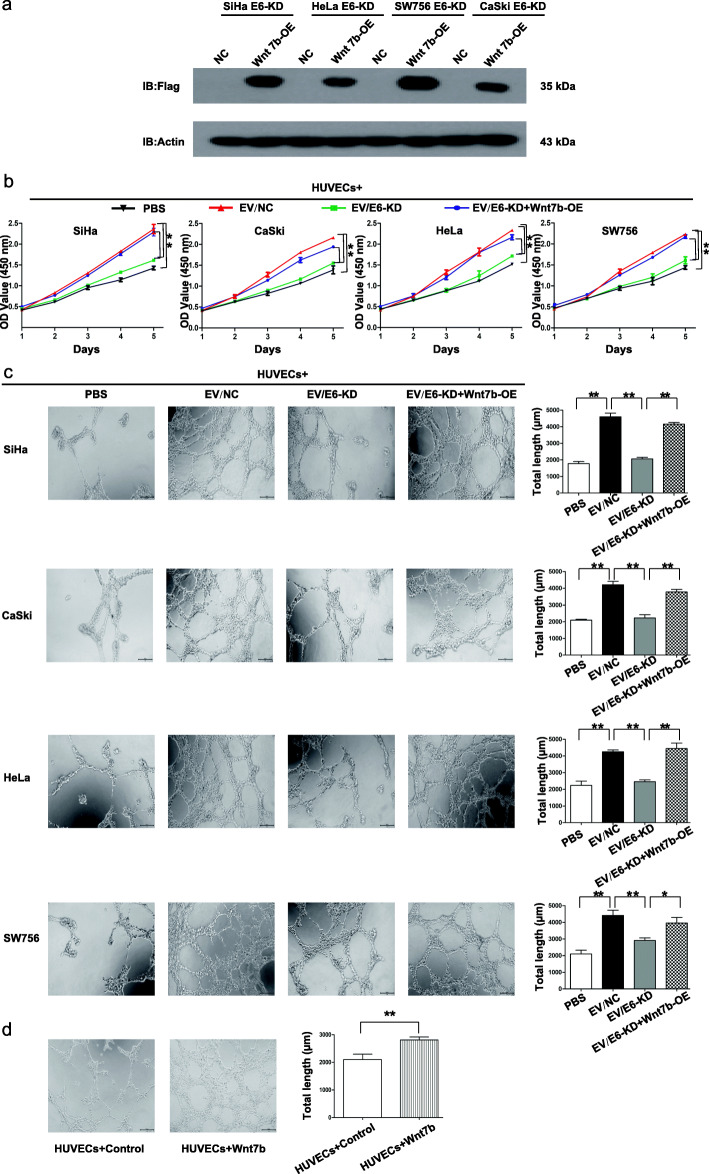
Extracellular vesicular Wnt7b mediates the HPV 16/18 E6-induced proliferation and angiogenesis of HUVECs. **a** After the introduction of Wnt7b-OE into HPV 16 E6-KD SiHa and CaSki cells and HPV 18 E6-KD HeLa and SW756 cells, Western blot examination confirmed flag-tagged Wnt7b expression in HPV E6-KD + Wnt7b-OE cells. **b** A CCK8 proliferation assay was performed with HUVECs. HUVECs were separately cocultured with PBS, EV/NC, EV/E6-KD or EV/E6-KD + Wnt7b-OE derived from the 4 HPV 16/18-positive CC cell lines. **c** Tube formation by HUVECs was evaluated. HUVECs were separately cocultured with PBS, EV/NC, EV/E6-KD or EV/E6-KD + Wnt7b-OE derived from the 4 HPV 16/18-positive CC cell lines. Left: representative photographs illustrating the dynamics of the angiogenic behavior of HUVECs. Right: the relative tube lengths in HUVEC cultures. **d** HUVECs were cocultured with recombinant Wnt7b and the controls. The treatment with recombinant Wnt7b promoted HUVEC angiogenesis compared with the treatment of controls. * *P* < 0.05, ** *P* < 0.01

To subsequently determine whether EVs can mediate HPV 16/18 E6-induced proliferation and angiogenesis and, if so, whether this effect is dependent on Wnt7b, we first overexpressed flag-tagged Wnt7b in HPV 16/18 E6-KD cells and performed western blot using anti-flag antibody. The results showed that the flag antibody specifically binds to Wnt7b-flag, which confirmed the overexpression of Wnt7b in all the four HPV 16/18 E6-KD cells (Fig. 4a). The results of CCK8 and tube formation assays respectively revealed that, compared with PBS treatment, treatment with EV/NC derived from the four HPV 16/18-positive CC cell lines promoted the proliferation and angiogenesis of HUVECs, while EV/E6-KD treatment significantly inhibited the proliferation and angiogenesis of HUVECs. However, treatment with EV/E6-KD + Wnt7b-OE restored the HUVEC proliferation and angiogenesis inhibited by E6-KD (Figs. 4b and c). Indeed, the treatment with recombinant Wnt7b promoted HUVEC angiogenesis compared with the treatment of controls (Fig. 4d). Altogether, these findings not only suggest that EVs can mediate HPV 16/18 E6-induced proliferation and angiogenesis but also indicate that EV-Wnt7b is indirectly or directly responsible for HPV 16/18 E6-induced HUVEC proliferation and angiogenesis.

### EV-Wnt7b mediates the HPV 16/18 E6-induced proliferation and angiogenesis of HUVECs by activating β-catenin signaling

**Fig. 5:**
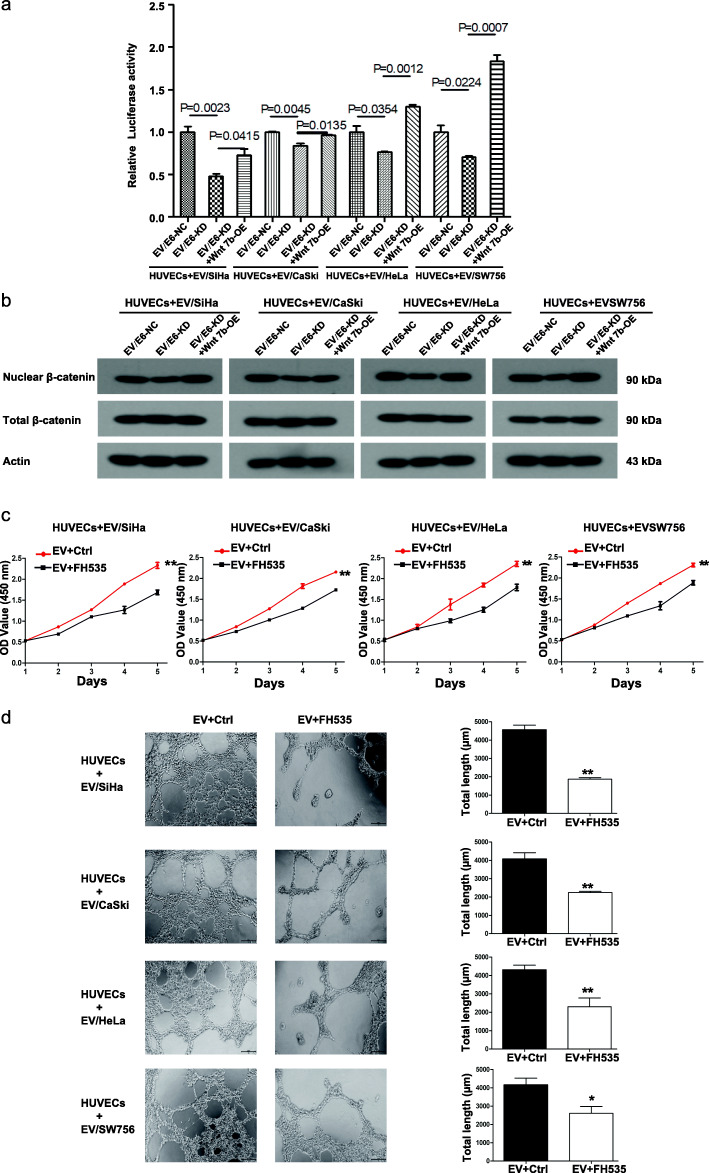
Extracellular vesicular Wnt7b mediates the HPV 16/18 E6-induced proliferation and angiogenesis of HUVECs through β-catenin signaling. **a** The transcriptional activity of β-catenin was assessed using the TOP/FOP dual-luciferase reporter system. The results showed that HUVECs treated with EV/E6-KD derived from the four HPV 16/18-positive CC cell lines exhibited less transcriptional activity of β-catenin than corresponding controls, while treatment with EV/E6-KD + Wnt7b-OE restored the transcriptional activity of β-catenin in HUVECs. **b** Western blot analysis of nuclear and total β-catenin expression in HUVECs treated with EV/NC, EV/E6-KD or EV/E6-KD + Wnt7b-OE derived from the 4 HPV 16/18-positive CC cell lines. **c** CCK8 proliferation assay with HUVECs. HUVECs were treated with EVs derived from the 4 HPV 16/18-positive CC cell lines in the presence or absence of FH535, an inhibitor of Wnt/β-catenin signaling. **d** Tube formation by HUVECs. HUVECs were treated with EVs derived from the 4 HPV 16/18-positive CC cell lines in the presence or absence of FH535. Left: representative photographs illustrating the dynamics of the angiogenic behavior of HUVECs. Right: the relative tube lengths in HUVEC cultures. * *P* < 0.05, ** *P* < 0.01

Since Wnt/β-catenin signaling is required for CC malignant transformation, we further determined whether β-catenin signaling is activated. The initial TOP/FOP dual-luciferase reporter analysis showed that HUVECs treated with EV/E6-KD derived from the four HPV 16/18-positive CC cell lines exhibited less transcriptional activity of β-catenin than corresponding controls, while treatment with EV/E6-KD + Wnt7b-OE restored the transcriptional activity of β-catenin in HUVECs (Fig. 5a). Moreover, the western blotting analysis also revealed that HUVECs treated with EV/E6-KD derived from the four HPV 16/18-positive CC cell lines exhibited less nuclear β-catenin expression than corresponding controls, while treatment with EV/E6-KD + Wnt7b-OE restored the nuclear translocation of β-catenin in HUVECs (Fig. 5b). These results confirm the transcriptional activation of β-catenin signaling.

To further explore the roles of Wnt/β-catenin signaling in the HPV 16/18 E6-induced proliferation and angiogenesis of HUVECs, we treated HUVECs with FH535, an inhibitor of Wnt/β-catenin signaling. The results of CCK8 and tube formation assays showed that treatment with FH535 abolished EV-mediated HPV 16/18 E6-induced HUVEC proliferation and angiogenesis, respectively (Figs. 5c and d). These results suggest that EV-Wnt7b activates β-catenin signaling in HUVECs, which is required for HPV 16/18-E6-induced cell proliferation and angiogenesis.

### EV-Wnt7b mediates HPV E6-induced CC tumor progression through β-catenin signaling in vivo

**Fig. 6:**
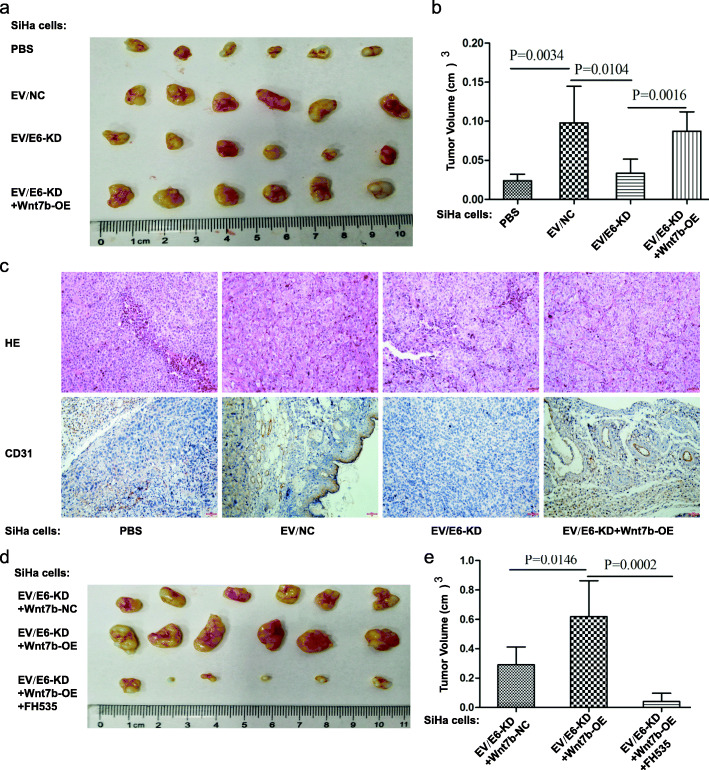
Extracellular vesicular Wnt7b mediates HPV E6-induced CC tumor progression through β-catenin signaling in vivo. **a** Representative images of tumor progression in mice from 4 groups (the mice were injected with PBS, EV/NC, EV/E6-KD or EV/E6-KD + Wnt7b-OE) were shown. **b** The final tumor volume of the mice in the 4 groups (PBS, EV/NC, EV/E6-KD and EV/E6-KD + Wnt7b-OE) was determined after the mice were euthanized. **c** In vivo angiogenesis was assessed by CD31 immunostaining. Haematoxylin and eosin-stained images and immunohistochemistry analysis of CD31 protein levels in xenograft tumor tissues were shown (bar: 100 μm). The results showed that the density of blood vessels (assessed via CD31 staining) was decreased in the EV/E6-KD tumours compared with the EV/NC tumours, while Wnt7b-OE abrogated the inhibition of tumor angiogenesis by EV/E6-KD. **d** Representative images of tumor progression in mice from 3 groups (the mice were injected with EV/E6-KD, EV/E6-KD + Wnt7b-OE, or EV/E6-KD + Wnt7b-OE+ FH535) were shown. **e** The final tumor volume of the mice in the 3 groups (EV/E6-KD, EV/E6-KD + Wnt7b-OE, and EV/E6-KD + Wnt7b-OE+ FH535) was determined

To further confirm the effects of HPV E6-regulated EV-Wnt7b on CC development in vivo, we injected SiHa cells subcutaneously to construct a xenograft model. Then, we injected PBS, EV/NC, EV/E6-KD or EV/E6-KD + Wnt7b-OE into the center of the xenograft tumors. Compared with PBS treatment, treatment with EV/NC promoted tumor growth, while EV/E6-KD treatment inhibited tumor growth. However, Wnt7b-OE abrogated the inhibition of tumor growth by EV/E6-KD (Figs. 6a and b). Additionally, the density of blood vessels (assessed via CD31 staining) was decreased in the EV/E6-KD tumours compared with the EV/NC tumours, while Wnt7b-OE abrogated the inhibition of tumor angiogenesis by EV/E6-KD (Fig. 6c). These findings suggest that EV-Wnt7b mediates HPV E6-induced CC tumor growth and angiogenesis in vivo, which is consistent with the in vitro results. Moreover, we also noticed that Wnt7b-OE in the E6-KD group promoted tumor growth; however, this promotion was abrogated by FH535 treatment (Figs. 6d and e). All these observations indicate that EV-Wnt7b mediates HPV E6-induced CC tumor progression through β-catenin signaling in vivo.

### Circulating EV-Wnt7b is correlated with aggressive clinical features and predicts a poor prognosis in CC

**Fig. 7:**
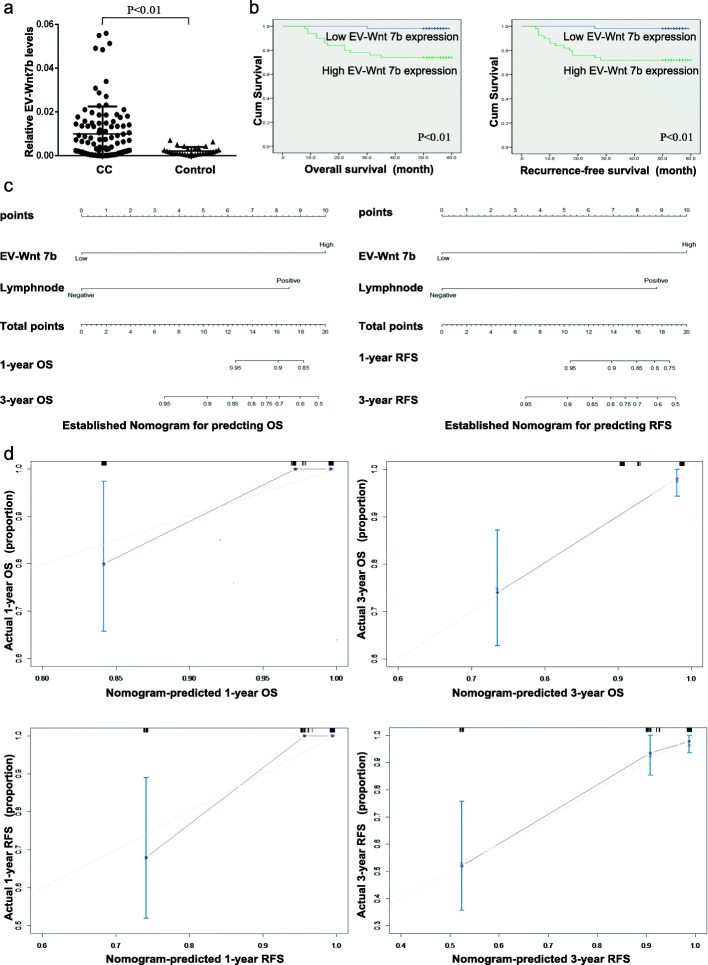
Circulating extracellular vesicular Wnt7b is correlated with aggressive clinical features and predicts a poor prognosis in CC. **a** Wnt7b expression in serum EVs from CC patients and controls. **b** Kaplan-Meier survival analysis of the OS and RFS rates of patients expressing high or low levels of extracellular vesicular Wnt7b. **c** A predictive nomogram model for the OS and RFS of CC patients according to serum extracellular vesicular Wnt7b and lymph node metastasis status. **d** The calibration curves of the nomogram for predicting 1-year and 3-year OS and RFS. The dashed line shows an ideal nomogram, and the solid line indicates the performance of the actual nomogram. The predicted observations matched the actual observations.

Considering the important roles of EV-Wnt7b in CC proliferation and angiogenesis, we further determined whether it has potential clinical value. As shown in Fig. 7a and Table 1, elevated serum EV-Wnt7b levels were observed in CC patients and significantly associated with relatively deep stromal invasion, lymphovascular invasion and lymph node metastasis. Additionally, patients with high levels of serum EV-Wnt7b had shorter OS and RFS than those with low levels (Fig. 7b and Table 2). Moreover, multivariate analysis demonstrated that serum EV-Wnt7b, independent of lymph node metastasis, was an independent prognostic factor for CC prognosis, including both OS and RFS (Table 3). Notably, a predictive nomogram for OS and RFS utilizing serum EV-Wnt7b and lymph node metastasis was successfully constructed (Fig. 7c). The c-indexes for the model to predict OS and RFS were 0.829 (95% CI: 0.738–0.920) and 0.837 (95% CI: 0.753–0.921), respectively. The calibration curves showed good prediction potential for 1- and 3-year OS and RFS (Fig. 7d). Altogether, these results suggest that serum EV-Wnt7b is associated with an aggressive phenotype and has the potential to be a predictive biomarker for CC patient survival.

**Table 1:** The correlations between the expression of serum EV-Wnt7b and clinicopathological factors of SCC patients

Variable	Low expression of serum EV-Wnt7b (***n*** = 51)	High expression of serum EV-Wnt7b (***n*** = 50)	***P***
Age, y (mean ± SD)			0.203
< =45	17	11	
> 45	34	39	
Stage			0.617
I	37	34	
II	14	16	
Tumor size			0.115
< =4 cm	39	31	
> 4 cm	12	19	
Lymphovascular invasion			< 0.001
Negative	33	10	
Positive	18	40	
Stromal invasion depth			0.028
< 1/2	24	13	
> =1/2	27	37	
Lymph node metastasis			< 0.001
Negative	45	25	
Positive	6	25	
Parametrial invasion			0.269
Negative	49	45	
Positive	2	5	
Margin			0.984
Negative	49	48	
Positive	2	2

**Table 2:** The correlations between clinicopathological characteristics and overall survival or recurrence-free survival of SCC patients using the Kaplan-Meier method

Variable	Overall Survival (months) Mean ± SE	***P***	Recurrence- Free Survival (months) Mean ± SE	***P***
Age, y (mean ± SD)		0.073		0.058
< =45	57.21 ± 1.75		57.07 ± 1.89	
> 45	52.75 ± 1.87		51.32 ± 2.12	
Stage		0.060		0.107
I	56.18 ± 1.41		55.03 ± 1.69	
II	48.17 ± 3.31		47.27 ± 3.60	
Tumor size		0.098		0.170
< =4 cm	55.56 ± 1.61		54.41 ± 1.87	
> 4 cm	50.55 ± 2.94		49.61 ± 3.21	
Lymphovascular invasion		0.023		0.052
Negative	57.05 ± 1.36		55.68 ± 1.86	
Positive	51.48 ± 2.25		50.53 ± 2.48	
Stromal invasion depth		0.067		0.058
< 1/2	57.60 ± 1.67		57.28 ± 1.89	
> =1/2	51.52 ± 2.00		50.11 ± 2.25	
Lymph node metastasis		< 0.001		< 0.001
Negative	57.71 ± 0.74		57.49 ± 0.86	
Positive	44.29 ± 3.86		41.29 ± 4.28	
Parametrial invasion		0.228		0.027
Negative	56.65 ± 1.47		54.06 ± 1.62	
Positive	46.86 ± 6.65		39.86 ± 7.81	
Margin		0.531		0.573
Negative	54.41 ± 1.48		53.34 ± 1.67	
Positive	48.25 ± 7.58		47.00 ± 8.66	
Serum EV-Wnt 7b		< 0.001		< 0.001
Low	58.43 ± 0.56		58.35 ± 0.64	
High	49.00 ± 2.69		46.90 ± 3.02

**Table 3:** Univariate and multivariate Cox regression analyses of the overall and recurrence-free survival of SCC patients

Variable	Recurrence-Free Survival				Overall survival			
	Univariate analysis		Multivariate analysis		Univariate analysis		Multivariate analysis	
	HR (95%CI)	***P***	HR (95%CI)		HR (95%CI)	***P***	HR (95%CI)	***P***
Age	5.649 (0.743 ± 42.966)	0.094	2.407 (0.287 ± 20.206)	0.592	5.249 (0.687 ± 40.135)	0.110		
Stage	2.246 (1.814 ± 6.196)	0.118			2.625 (0.920 ± 7.486)	0.071	1.763 (0.585 ± 5.310)	0.314
Tumor size	2.001 (0.726 ± 5.519)	0.180			2.344 (0.822 ± 6.684)	0.111		
Lymphovascular invasion	3.250 (0.917 ± 11.519)	0.068	0.324 (0.064 ± 1.631)	0.171	4.802 (1.074 ± 21.458)	0.040	0.414 (0.080 ± 2.141)	0.293
Stromal invasion depth	0.263 (0.059 ± 1.168)	0.079	0.724 (0.150 ± 3.503)	0.689	0.272 (0.061 ± 1.217)	0.089	0.885 (0.168 ± 4.667)	0.885
Lymph node metastasis	11.448 (3.223 ± 40.664)	0.000	7.695 (1.562 ± 37.900)	0.012	10.273 (2860 ± 36.893)	0.000	7.156 (1.415 ± 36.176)	0.017
Parametrial invasion	2.165 (0.488 ± 9.595)	0.309			2.433 (0.544 ± 10.872)	0.245		
Margin	1.773 (0.233 ± 13.492)	0.580			1.892 (0.247 ± 14.469)	0.539		
Serum EV-Wnt 7b	16.554 (2.176 ± 125.962)	0.007	11.579 (1.297 ± 103.400)	0.028	15.159 (1.982 ± 115.93)	0.009	11.450 (1.289 ± 101.735)	0.029

## Discussion

Although persistent infection with HPV 16/18 is regarded as the most important cofactor for CC progression [2, 3], it is insufficient on its own. Emerging evidence has indicated that the TME is a critical contributor to cancer progression [6–10]. In the TME, cancer cells can remodel their unfavorable microenvironment to support continuous and sustained proliferation, angiogenesis and metastasis [6–10]. Therefore, investigations of the detailed cellular and molecular events, especially how HPV 16/18 mediate cancer-TME communication and reshape the TME, are urgently needed. In the present study, our in vitro and in vivo experiments revealed that EVs mediated crosstalk between HPV 16/18-positive CC cells and HUVECs to remodel the TME for HPV E6-induced CC progression. By means of EVs, E6-regulated Wnt7b mRNA could be transported from HPV 16/18-positive CC cells to recipient HUVECs and then modulate the HUVECs to take on a more proliferative and proangiogenic phenotype by acting on the β-Catenin signaling pathway.

EVs are increasingly recognized as critical contributors in cancer progression. They are potent mediators of communication between cancer cells and their surrounding microenvironment, which contains various types of host cells, including HUVECs [16–23, 35]. Given that E6 oncoproteins are the critical drivers of HPV 16/18-associated CC development [36, 37], we hypothesized that HPV 16/18 E6 influences the TME via EVs. As expected, we observed that EVs derived from the four HPV 16/18 E6-KD cell lines and corresponding control cells were effectively internalized by HUVECs. Moreover, compared with those of HUVECs treated with EV/NC, the proliferative and proangiogenic abilities of HUVECs treated with EV/E6-KD were significantly inhibited both in vitro and in vivo. These findings suggest that EVs serve as important messengers between HPV 16/18-positive CC cells and HUVECs. Via EVs, the HPV E6 oncogene could render recipient HUVECs to take on a more proliferative and proangiogenic phenotype for CC progression.

The cargos, including proteins, DNAs, RNAs, and signaling molecules, contained in EVs are known to be transported between cells and to be responsible for EV function [19–23]. As classic signaling molecules, EV-shuttled Wnt signaling molecules, such as Wnt 11, Wnt3a, Wnt5b, and Wnt4, have been reported to be involved in cancer progression [27–30]. In the present study, we noticed that Wnt7b mRNA was highly expressed in the 4 HPV 16/18-positive CC cell lines and their EVs compared with HPV-negative cells and their EVs, respectively. By the way, we also tested Wnt7a mRNA expression in CC cells and found that Wnt7a mRNA was absent in Hela cells and exhibited very low expression in Siha cells (data not shown), which is consistent with a previous study [38]. Additionally, we observed that Wnt7b mRNA and protein can be downregulated by both HPV 16 and 18-E6 knockdown. By means of EVs, E6-regulated-Wnt7b mRNA can be transported from HPV 16/18-positive CC cells to HUVECs. Moreover, we overexpressed Wnt7b in 4 HPV 16/18 E6-KD cell lines and isolated their EVs. The further western blotting analysis, CCK8 and tube formation analysis showed that Wnt7b mRNA transferred by EVs in HUVEC cells might be followed by Wnt7b protein synthesis in the cells, which may consequently exert effect on the proliferation and angiogenesis of HUVECs, finally contributing to HPV 16/18 E6-induced CC proliferation and angiogenesis. Admittedly, we cannot exclude the partial transportation of Wnt7b protein to HUVECs. The in vivo results further confirmed that EV-Wnt7b is indirectly or directly responsible for HPV 16/18 E6-induced HUVEC proliferation and angiogenesis.

Accumulating evidence has demonstrated that Wnt/β-catenin signaling is required for malignant cancer transformation [39, 40]. Once Wnt signaling is activated, β-catenin accumulates and is stabilized in the cytoplasm and translocates into the nucleus to act as a cofactor in regulating the transcription of various genes involved in cancer progression [39, 40]. Inspired by this knowledge, we therefore tested the activation of β-catenin signaling in HUVECs. We found that EV/E6-KD-treated HUVECs exhibited both the less transcriptional activity of β-catenin and nuclear β-catenin expression than corresponding controls, while treatment with EV/E6-KD + Wnt7b-OE restored both the transcriptional activity of β-catenin and the nuclear translocation of β-catenin in HUVECs. These results confirm the activation of Wnt/β-catenin signaling. Moreover, in vitro results showed that treatment with FH535, an inhibitor of Wnt/β-catenin signaling [41] abolished EV-mediated HPV E6-induced HUVEC proliferation and angiogenesis. Further in vivo results confirmed that the treatment with FH535 abolished HPV E6-induced CC growth. Collectively, these results suggest that EV-Wnt7b can mediate HPV E6-induced CC progression by acting on the β-catenin signaling pathway.

Currently, emerging evidence suggests that EV-RNAs are appealing biomarkers for cancer diagnosis and prognosis because they are stable, shielded against RNase degradation and noninvasive [42, 43]. Considering the critical roles of EV-Wnt7b in CC proliferation and angiogenesis, we raised the possibility that circulating EV-Wnt7b might serve as a potential biomarker for CC prognosis. As expected, serum EV-Wnt7b levels were elevated in CC patients and significantly related to an aggressive CC phenotype. Moreover, survival analyses revealed that elevated serum EV-Wnt7b levels were related to reduced OS and RFS and that serum EV-Wnt7b is an independent prognostic factor for both OS and RFS. Notably, we successfully established a novel predictive nomogram utilizing serum EV-Wnt7b, which further confirmed that serum EV-Wnt7b had the potential to be a predictive biomarker for CC prognosis.

A major limitation of this study was that the sample size was small. Therefore, a multicenter, large-sample study should be performed to verify the predictive value of serum EV-Wnt7b in CC prognosis. In addition, the possibility that Wnts expression may be cell type specific cannot be ruled out, which requires a future deeper exploration. Further, our future studies should also focus on exploring the exact molecular network of the “HPV E6 / EV-Wnt7b / β-catenin” pathway. Moreover, whether the known “HPV E6 / p53” pathway interacts with the “HPV E6/EV-Wnt7b/β-catenin” pathway will be a meaningful area for further exploration.

**Fig. 8:**
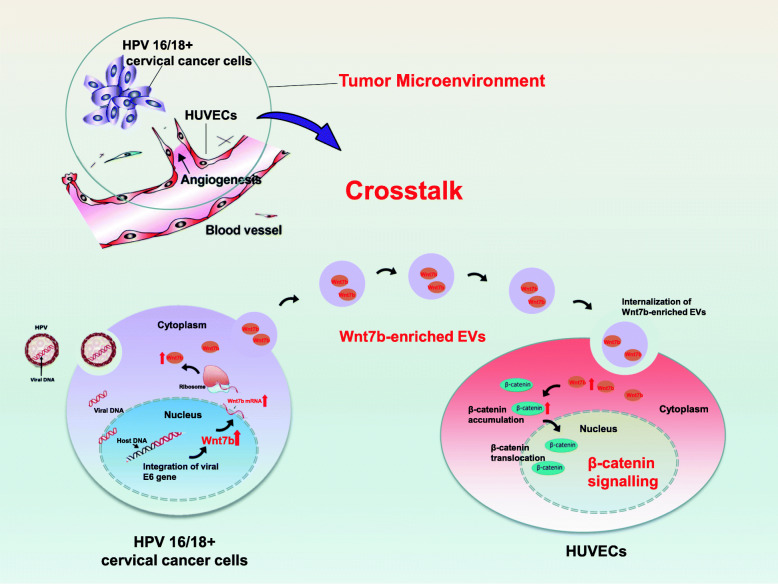
Schematic diagram depicting the proposed mechanism for HPV 16/18 E6-induced CC progression from the perspective of “EV-shuttled Wnt7b acts on β-catenin signaling”. In the TME, HPV 16/18 E6 upregulated Wnt7b expression in both HPV 16/18-positive CC cells and their EVs. These Wnt7b mRNA-enriched EVs could be transferred to and internalized by recipient HUVECs and then modulate HUVECs toward more proliferative and proangiogenic behaviors by acting on β-catenin signaling, eventually facilitating CC progression

In conclusion, our study is the first to indicate that EVs mediate crosstalk between HPV 16/18-positive CC cells and HUVECs to remodel the TME for HPV 16/18 E6-induced CC progression. The proposed detailed novel mechanism is as follows: in the TME, HPV 16/18 E6 upregulated Wnt7b mRNA expression in both HPV 16/18-positive CC cells and their EVs. These Wnt7b mRNA-enriched EVs could be transferred to and internalized by recipient HUVECs, followed by Wnt7b protein synthesis in the cells and then modulate the HUVECs toward more proliferative and proangiogenic behaviors by acting on β-catenin signaling (Fig. 8). Additionally, elevated serum EV-Wnt7b was correlated with an aggressive clinical phenotype. Based on the novel predictive nomogram model we established, we propose that serum EV-Wnt7b may have potent potential as a predictor for CC prognosis.

## Supplementary Information

**Additional file 1 :Supplementary Fig. 1**. Wnt7b protein in CC cells and their centrifuged EVs-free conditioned medium. Western blot analysis showed that Wnt7b protein was absent or with very low expression in the centrifuged EVs-free conditioned medium, but with significantly high expression in all the four HPV 16/18-positive cell lines compared to HPV-negative C33A cells.

**Additional file 2: Supplementary Table 1**. Baseline characteristics of patients with cervical cancer. **Supplementary Table 2**. Primer sequences of the studied genes.

## Data Availability

The data and materials used or analysed during the current study are available.

## Abbreviations

EVs: Extracellular vesicles
HPV: Human papillomavirus
CC: Cervical cancer
TME: Tumor microenvironment
HUVECs: Human umbilical vein endothelial cells
OS: Overall survival
RFS: Recurrence-free survival
KD: Knockdown
OE: Overexpression
qRT-PCR: Quantitative real-time polymerase chain reaction
