# Supplementary figures and images for: Extracellular vesicular Wnt7b mediates HPV E6-induced cervical cancer angiogenesis by activating the β-catenin signaling pathway
Source: J Exp Clin Cancer Res. 2020 Nov 25;39:260. doi: 10.1186/s13046-020-01745-1 (PMC7687741; doi:10.1186/s13046-020-01745-1)

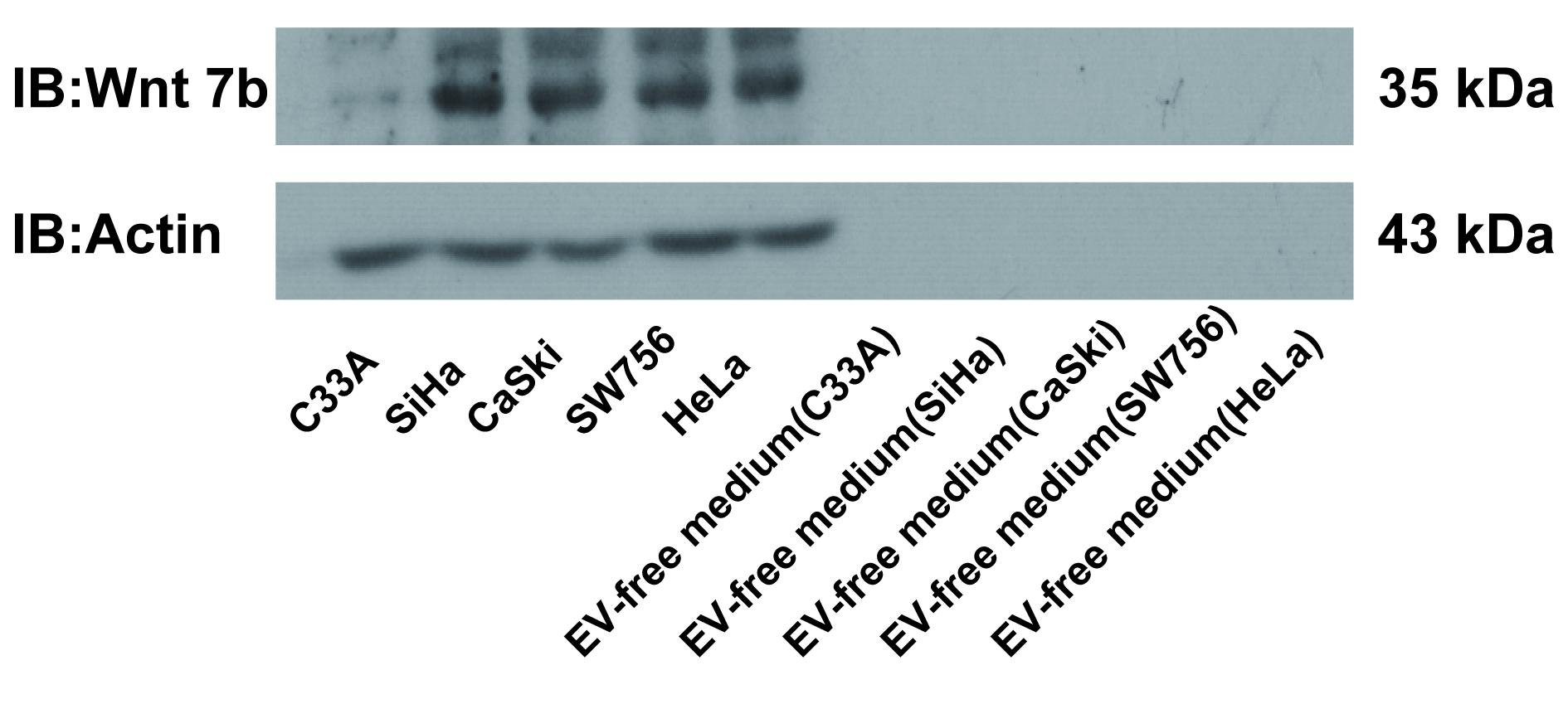

Supplement: Supplementary file 1 — Additional file 1 :Supplementary Fig. 1. Wnt7b protein in CC cells and their centrifuged EVs-free conditioned medium. Western blot analysis showed that Wnt7b protein was absent or with very low expression in the centrifuged EVs-free conditioned medium, but with significantly high expression in all the four HPV 16/18-positive cell lines compared to HPV-negative C33A cells. [file 13046_2020_1745_MOESM1_ESM.tif]
