# Supplementary material for: Extracellular vesicular Wnt7b mediates HPV E6-induced cervical cancer angiogenesis by activating the β-catenin signaling pathway
Source: J Exp Clin Cancer Res. 2020 Nov 25;39:260. doi: 10.1186/s13046-020-01745-1 (PMC7687741; doi:10.1186/s13046-020-01745-1)
Supplement: Supplementary file 2 — Additional file 2: Supplementary Table 1. Baseline characteristics of patients with cervical cancer. Supplementary Table 2. Primer sequences of the studied genes. [file 13046_2020_1745_MOESM2_ESM.docx]

**Supplementary Table 1. Baseline characteristics of patients with cervical cancer**

| **Variable** | **Number (%)** |
| --- | --- |
| Age, y (mean ± SD) | 50.84±9.93 |
| <=45 | 28(27.7%) |
| >45 | 73 (72.3%) |
| Stage |  |
| IA2 | 4 (4.0%) |
| IB1-IB2 | 68 (67.3%) |
| IIA1-IIA2 | 29 (28.7%) |
| Tumor size |  |
| <=4cm | 70(69.3%) |
| >4cm | 31(30.7%) |
| Lymphovascular invasion |  |
| Negative | 43(42.6%) |
| Positive | 58(57.4%) |
| Stromal invasion depth |  |
| <1/2 | 37(36.6%) |
| >=1/2 | 64(63.4%) |
| Lymph node metastasis |  |
| Negative | 70(69.3%) |
| Positive | 31(30.7%) |
| Parametrial invasion |  |
| Negative | 94(93.1%) |
| Positive | 7(6.9%) |
| Margin |  |
| Negative | 97(96.0%) |
| Positive | 4(4.0%) |

**Supplementary Table 2 Primer sequences of the studied genes.**

| **Gene** | **Primer** | **Sequence (5'to3')** |
| --- | --- | --- |
| Wnt1 | Forward | CTCTTCGGCAAGATCGTCAACC |
|  | Reverse | CGATGGAACCTTCTGAGCAGGA |
| Wnt2 | Forward | AGGATGCCAGAGCCCTGATGAA |
|  | Reverse | AGCCAGCATGTCCTGAGAGTAC |
| Wnt3a | Forward | ATGAACCGCCACAACAACGAGG |
|  | Reverse | GTCCTTGAGGAAGTCACCGATG |
| Wnt4 | Forward | GCTGGAGAAGTGCGGCTGTGA |
|  | Reverse | CCACAAACGACTGTGAGAAGGC |
| Wnt5a | Forward | TACGAGAGTGCTCGCATCCTCA |
|  | Reverse | TGTCTTCAGGCTACATGAGCCG |
| Wnt6 | Forward | GTGCAACTGCACAACAACGAGG |
|  | Reverse | GAAATGGAGGCAGCTTCTGCCA |
| Wnt7b | Forward | AGAAGACCGTCTTCGGGCAAGA |
|  | Reverse | AGTTGCTCAGGTTCCCTTGGCT |
| Wnt10b | Forward | CTCGGGATTTCTTGGATTCCAGG |
|  | Reverse | GCCATGACACTTGCATTTCCGC |
| Wnt11 | Forward | CTGTGAAGGACTCGGAACTCGT |
|  | Reverse | AGCTGTCGCTTCCGTTGGATGT |
| HPV16-E6 | Forward | GTTTCAGGACCCACAGGAGC |
|  | Reverse | CGTCGCAGTAACTGTTGCTTG |
| HPV18-E6 | Forward | CCAGAAACCGTTGAATCCAGC |
|  | Reverse | AGTCGTTCCTGTCGTGCTC |
| GAPDH | Forward | GTCTCCTCTGACTTCAACAGCG |
|  | Reverse | ACCACCCTGTTGCTGTAGCCAA |
